# User-Cell Association for Security and Energy Efficiency in Ultra-Dense Heterogeneous Networks

**DOI:** 10.3390/s21020508

**Published:** 2021-01-13

**Authors:** Dania Marabissi, Lorenzo Mucchi, Simone Morosi

**Affiliations:** Department of Information Engineering, University of Florence, I-50139 Florence, Italy; dania.marabissi@unifi.it (D.M.); simone.morosi@unifi.it (S.M.)

**Keywords:** physical-layer security, ultra dense networks, energy efficiency

## Abstract

The last decades have been characterized by an exponential increase in digital services. The demand is foreseen to further increase in the next years, and mobile networks will have to mandatorily supply connections to enable digital services with very different requirements, from ultra high speed to ultra low latency. The deployment and the coexistence of cells of different size, from femto to macro, will be one of the key elements for providing such pervasive wireless connection: the ultra dense networks (UDN) paradigm. How to associate users and base stations is one of the most investigated research topics. Many criteria can be drawn, from minimization of power consumption to optimization of throughput. In this paper we propose a new utility to optimize two of the most important features of future mobile connection: security and energy consumption. By using our utility it is possible to jointly select the base station to be activated in a UDN, and associate users to the base stations with the aim of maximizing the secure throughput by spending the minimum energy. Moreover, we propose a heuristic that allows to achieve performance very close to the optimal one with reduced complexity. Effectiveness of the proposed approach is proved by means of comparison with benchmark approaches.

## 1. Introduction

The last decades have been characterized by an unprecedented fast development of wireless technologies. The 5th generation (5G) of mobile network is nowadays being deployed and 6G is under investigation. Pervasive wireless connection is mandatory to meet the increasing demand of digital services, during the everyday life of citizens [1]. In order to meet the goal of a pervasive connectivity and to provide services with different constraints (from ultra low latency to ultra high speed), cells of different size and with different features should coexist and collaborate: the paradigm of ultra dense network (UDN) [2]. UDN is considered one of the best ways to meet user expectations and support future wireless services development [3].

The more the services on-demand and on-mobility, anytime-anywhere, the more the amount of information in the air, often critical from the point of view of privacy, but not only. The data which every minute flows in the wireless channel can be very critical, from body/health information to vehicles assisted/autonomous driving or industrial automation. The word “smart” is nowadays pre-imposed to several key-words: factory, farm, vehicle, body, etc. One of the main assumptions under this word is the always-on wireless connectivity, since smartness implies flow of information, often in real-time. Unfortunately, the benefit of wireless connectivity is also an issue: the channel can be heard by any receiver, legitimate or not. In order to trust the digital services of the future, security must be inserted as a key feature, from the very beginning of the design of the wireless system/technology [4]. physical-layer security (PLS) is envisioned as a promising technique to provide an additional level of security, since it does not rely on the assumption of limited computational power of the attacker, as the classical cryptography does. While in cryptography, a message is correctly taken by the attacker from the wireless channel but its meaning cannot be interpreted, in PLS the attacker is not able to correctly detect any message by analyzing the channel [5].

There is another critical issue that all approaching technologies should seriously take into account: the energy consumption. The Information and Communication Technology (ICT) sector is one of the main contributors to the increase of CO${}_{2}$ level on Earth [6]. A conservative estimation currently puts around 4% of all electricity consumption and over 2% of all CO${}_{2}$ emissions as the result of ICT use. If 6G-like on-demand on-mobility services are added, the consumption numbers are envisioned to double [7]. It is extremely important for the success of both 5G and 6G services to intrinsically provide minimum consumption, i.e., to meet the energy efficiency by design. 6G is the first mobile technology which is evaluating to directly impose energy efficiency constraints (Terabyte/Joule) to move information.

Energy efficiency and security are, thus, two of the key issues that next generation wireless connectivity has to face. This is particularly true for UDNs. Indeed UDNs offer new opportunities for achieving PLS because wireless channels are more random and inter-cell interference can be beneficial. Using suitable interference management systems, the legitimate user can benefit form network densification while the eavesdropper experiences strong interference that degrades its signal quality [8]. Moreover, cells’ densification allows to provide coverage with low-power access points; thus, if the cells are suitably activated depending the traffic on a given area, UDNs can be a mean to improve energy efficiency [9].

It is important to point out that the upcoming 5G and the future 6G communications will have to cope with an extremely large set of objectives and figures of merit, such as enhanced throughput, very short latency, a generalized coverage with stable user experience for any possible speed of the devices, unprecedented spectral and energy efficiencies, and so on. These differentiated goals end up mutually influencing each other so that it is impossible to find a general optimization rule. As a result, new multi-objective optimization strategies have been recently introduced and developed [10,11,12]. The lack of a general optimization strategy and the pursuit of an adequate tradeoff between conflicting objectives can be seen as two major research goals also for the problem that is considered in this paper: security and energy efficiency have been largely treated separately so far; nonetheless, a joint optimization is usually better, in order to meet an equilibrium between opposite requirements.

Often it is not taken into account how much more energy is consumed to provide secure services, and, similarly, the network is configured to provide maximum quality of service (QoS), not considering energy consumption or security. Resource allocation between heterogeneous networks is one of the main key drivers to provide QoS to users, but it can also be exploited to provide energy efficiency as well as security. The aim of this paper was to study resource allocation strategies to provide security and energy efficiency at the same time. In particular, we propose a new strategy of association between user terminals and small base stations (from femto to microcells) that provides the maximum secure throughput for the user and the minimum energy consumption suitably selecting which cells must be turned off.

### 1.1. State-of-the-Art

In PLS, a non-zero secure rate can be obtained by any strategy that produces an advantage in terms of signal-to-noise ratio (SNR) over the attacker. This opened the research to study different strategies, from transmit power optimization to artificial noise injection.

The joint optimization of the secrecy rate and the total consumed power was studied in [13]. Therein, the instantaneous received SNR for the legitimate terminal is assumed to be strictly larger than the eavesdropper receiver. The extension to multi-antenna scheme (MIMO) is investigated in [14], considering the downlink. In [15], a power allocation policy was developed to maximize the secrecy information rate while maintaining the harvested energy requirement of the energy receiver.

The joint optimization of secrecy and energy efficiency has been recently investigated in Energy Harvesting scenarios. In [16,17], the maximization of the secrecy energy efficiency (SEE) is obtained by means of PLS-based signal processing. In [12], a multi-objective optimization problem is targeted to full-duplex (FD) networks with simultaneous wireless information and power transfer (SWIPT).

In [18], an energy-efficient relay selection scheme which jointly considers best relay selection and dynamic power allocation in order to maximize the secrecy rate as well as to minimize energy consumption is provided. The role of the interference in providing PLS in (downlink only) internet of things (IoT) is considered in [19], where also the cost in terms of energy is calculated. In [20], a radio resource allocation framework to optimize both the confidentiality and the energy efficiency of a communication system is proposed. In [21], cross-layer cooperation as a viable solution for the achievement of reliability and energy efficiency in wireless communication is proposed. A survey on energy efficient design of wireless networks can be found in [22].

For what concerns the cell-user association, in the traditional approach the user terminal (UE) selects the base station (BS) that provides the highest signal-to-noise plus interference ratio (SINR). Authors in [23,24] propose optimization models for wireless network design under SIR and users-to-BS association constraints. Other approaches involve a meta-heuristic approach for a robust association that also takes into account the uncertainty of data rate transmissions [25]. Application to sensors can be found in [26], where an optimal user association strategy for large-scale Internet-of-Things (IoT) sensor networks is illustrated.

Only a few papers consider network security. In [27], the UE selects the BS that provides the highest secrecy rate, but inter-cell interference is not considered, while it is a basic element of UDNs. Differently in [28], in-band interference is considered and the maximum secrecy rate association is approximated using the Maclaurin formulation. In [29], the maximum secure area is considered. There are also papers that consider energy efficiency as association policy as in [30,31,32,33]. In [34], the transmission power is minimized by minimizing the number of active small-cells with a constraint on the minimum achievable rate. The complexity of the problem and a heuristic solution are evaluated. Finally, in [35,36] the relations between secrecy capacity and energy consumption are investigated in heterogeneous cellular networks and original metrics that bind the secure area of a cell, the afforded date-rate and the power spent by the BS are proposed as a tool for the evaluation of different joint optimization strategies.

### 1.2. Our Contribution

Having considered the aforementioned state-of-the-art, a study that comprises the use of user-BS association in ultra dense networks to integrate physical-layer security alongside taking care of the energy efficiency has not been conducted so far. The contribution of this paper can be thus summarized as follows:

- the investigation of the benefits of UE-BS association in improving selected performance indicators as well as minimizing energy consumption;
- the definition of a new utility function which integrates together the secure throughput per user and the energy consumption of the network. Thus, the cell activation and users’ association are jointly performed;
- proposal of a heuristic for user-BS association and cell-activation selection able to achieve performance close to the optimal one;
- the application of the above-mentioned utility to the use case of an ultra dense network;
- the comparison of the proposed utility with three other utilities known in literature.

The paper took into account mobile terminals, but the results are valid for any device able to connect to a cellular network. In the future cellular network (6G), the intelligence will be given to even small devices, such as sensors of any type, installed on the human body or in the environment or in portable objects (a bottle of water, etc.), or in moving objects (vehicles, drones, etc.), together with classical portable devices (smartphone, tablet, etc.). All these devices have to be constantly connected, and energy consumption as well as security are two of the main features to be absolutely guaranteed anytime-anywhere. Moreover, physical-layer security (PLS) is a promising mechanism to provide security to low-resourced devices like sensors, or, in general, when classical cryptography cannot be directly applied or to provide an additional level of security to the devices in the network. This paper proposes a metric which takes into account simultaneously the energy consumption and the physical-layer security provided to the user (in terms of secure throughput), based on the association between users and base stations. The term “user” can be interpreted in many ways, from a smartphone to, e.g., a wearable sensor.

## 2. System Model

**Figure 1:**
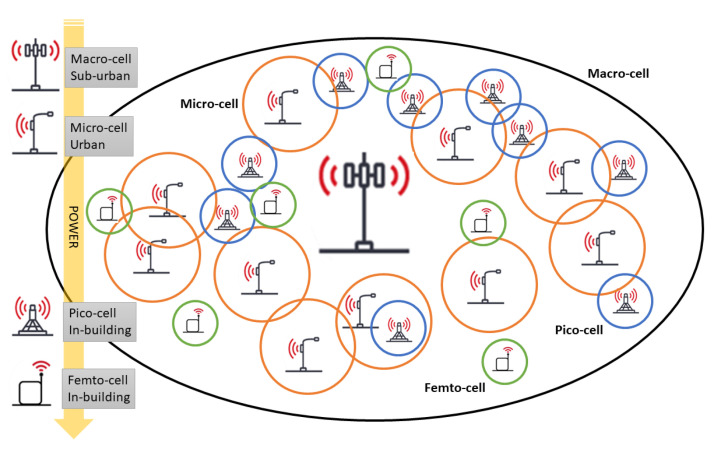
Sketch of UDN: macrocell with micro, pico, and femto cells.

Let us assume to have an ultra dense deployment of heterogeneous cells where a macrocell layer providing basic service and coverage is overlayed by an ultra-dense layer of small base stations (SBSs) with different characteristics (Figure 1).

Macrocell and small cells operate in different frequency bands, thus avoiding cross-tier interference. Conversely, SBSs reuse the same radio resources.

We consider two different scenarios:

- small-cell and macrocell layers serve two different classes of users, (i.e., users requiring secure communications are served by the small cells and not by the macrocell). Hence, the focus is only on the small-cell layer composed by micro, pico, and femto cells, generally indicated as small cells in what follows;
- users can be served by macrocells or small cells without any differentiation.

In both cases, small cells are randomly distributed in the considered area following a Poisson point process (PPP) distribution. The density of the PPP is represented by $\lambda_{S}$.

Users are supposed to be randomly distributed in the area *A* following an independent PPP distribution with density $\lambda_{U}$. Each user receives the reference signal broadcasted by the BSs around the mobile terminal. The user terminal can then evaluate and rank each signal-to-noise plus interference ratio (SINR) received over the sensitivity threshold.

The SINR received by user *u* from cell *c* can be written as

(1) $${\mathrm{SIN}R}_{u,c}=\frac{P_{c}^{\left[\mathrm{TX}\right]}{\left|h_{u,c}\right|}^{2}\rho_{u,c}}{\sigma_{u}^{2}+\sum_{\begin{array}{c} s=1\\ s\neq c \end{array}}^{N_{C}}{\left|h_{u,s}\right|}^{2}P_{s}^{\left[\mathrm{TX}\right]}}$$

where $P_{s}^{\left[\mathrm{TX}\right]}$ is the transmission power of the signal broadcasted by the *s*-th cell, $N_{C}$ is the total number of SBSs, $\rho_{u,s}$ is the path loss from the *s*-th SBS to the *u*-th UE, $\sigma_{u}^{2}$ is the noise power at the receiver, and $|h_{u,s}{|}^{2}$ represents the gain of the channel between the *s*-th cell and the *u*-th UE that follows an exponential distribution with unit mean.

The path loss model $\rho_{u,c}$ is assumed to be dual slope that takes into account also the line of sight component at very short distances from the BS and better models the effects of BSs densification [37]

(2) $$\rho_{u,c}\left(d_{u,c}\right)=\left\{\begin{array}{cc} K_{0}d_{u,c}^{-\alpha_{1}}, & d_{u,c}\leq \bar{d}\\ K_{1}d_{u,c}^{-\alpha_{2}}, & d_{u,c}>\bar{d} \end{array}\right.$$

where $d_{u,c}$ is the distance between the BS of cell *c* and the terminal of the user *u*, $\bar{d}$ is the critical distance that separates the close-in and the long-range path loss zones, $K_{0}$ is a catch-all constant that is equal to the path loss for unitary distance, $K_{1}$ is a constant to ensure continuity between the two path-loss regions, $\alpha_{1}$ is the close-in path loss exponent (usually equal to 2), and $\alpha_{2}$ is the long-range path loss exponent (usually ranging from 2 to 6),

Given the SINR expression, the capacity (normalized to the sub-band width) achievable by the *u*-th user if associated with the *s*-th SBS is

(3) $$C_{u,c}=log\left(1+SINR_{u,c}\right)$$

In this paper we investigate the user association problem. Each user can associate with a single cell depending on a specific utility function U. In particular, the *u*-th user selects the cell ${\hat{c}}_{u}$ that maximizes the utility $U_{u,c}$ so that

(4) $${\hat{c}}_{u}=\max_{c}U_{u,c}$$

The standard solution is that each user is associated with the cell providing the maximum SINR, that is $U_{u,c}=SINR_{u,c}$ defined in (1) [38]. However, in a UDN usually the UE is under the coverage of several BSs and the user-cell association may have a great impact on different performance metrics, and one of these can be the security. Association policies aiming at improving the network security have been investigated in [27]. The serving BS is selected as the one that provides the maximum secrecy rate in [27,28] while in [29] the maximum secure area.

In this paper, we focus on an new association policy that aims at optimizing security jointly with power consumption. Indeed, we can observe that being cells active or in idle state depending if they have or not users to serve, different association choices determine different patterns of active cells, that lead to different SINR/interference distributions in the considered area. This impacts both the security and the power consumption of the system.

The association policy we propose here is executed centrally, following the cloud/cent-ralized radio access (C-RAN) concept: a large number of access points are distributed over the coverage area and are connected to a central processing unit (CPU) that allows cooperation among cells. Hence, the CPU decides the pattern of activation of the cells, and then each user associates with the (active) cell that provides the highest utility. The CPU needs to know the users’ SINR that each user can derive from the synchronization signals periodically broadcasted by each BS. Then, the SINR measures are fed back to the BSs and forwarded to the CPU.

### Power Consumption Model

To evaluate the energy efficiency of the system, it is necessary to consider the total power consumption of each BS, namely *P*. The total power consumption of a generic *active BS* is composed by the radiated power, $P^{\left[\mathrm{TX}\right]}$, and by the power consumed for signal processing, power amplifiers, cooling systems, etc., namely $P_{0}$. Furthermore, even if on *idle mode*, the BS has a constant power consumption, $P_{idle}<P_{0}$. Referring to [39], the total power consumption of each BS can be expressed as

(5) $$P=\left\{\begin{array}{ccc} P_{0}+\Omega_{p}P^{\left[\mathrm{TX}\right]} & \mathrm{if} & 0<P^{\left[\mathrm{TX}\right]}\leq P_{max}\\ P_{idle} & \mathrm{if} & P^{\left[\mathrm{TX}\right]}=0 \end{array}\right.$$

where $\Omega_{p}$ is a scaling factor.

Different BS types have different parameters that depend on the specific implementation as detailed in Table 1.

## 3. Proposed Association Utility

In addition to the standard SINR utility for UE association, we define a new utility that takes into account also the security and the power consumption.

### 3.1. Security Metrics Definition

PLS defines a standard metric to measure how much information can be transferred in a wireless link without eavesdropping: the secrecy capacity $C_{u,s}^{\left[sec\right]}$ [40]. The secrecy capacity is defined as the difference between the capacity of the legitimate link $C_{u,c}$ (between the *u*-th UE and the *c*-th SBS) and the capacity of the eavesdropper $C_{E,c}\left(\hat{x},\hat{y}\right)$ in position $\left(\hat{x},\hat{y}\right)$. Thus, $C_{s}=\max\left\{0,C_{u,c}-C_{E,c}\left(\hat{x},\hat{y}\right)\right\}$. $C_{E,c}\left(\hat{x},\hat{y}\right)$ is the capacity of the link from transmitting cell *c* and the eavesdropper in position $\left(\hat{x},\hat{y}\right)$. The expression of the capacity is as in (3) where the SINR value is referred to Eve. This metric requires to know Eve’s location, which is not an information usually known in the real world. In order to drop this requirement, in [41] a secrecy capacity averaged over an area is proposed as a new metric.

Considering the above-mentioned results, we derive two security metrics:

- Secure Area $A_{u,c}^{\left[\sec\right]}$. The secure area is defined as the set of locations of an area where the capacity of the legitimate channel $C_{u,c}$ is strictly greater than the capacity of the eavesdropper (Eve) channel $C_{E,c}$. In other words, assuming that Eve is in a generic location (x,y)∈A then $C_{E,c}\left(x,y\right)<C_{u,c}$
  (6) $$A_{u,c}^{\left[\sec\right]}=\left\{\left(x,y\right)\in A\;\;\;|\;\;\;C_{u,s}^{\left[sec\right]}\left(x,y\right)=C_{u,c}-C_{E,c}\left(x,y\right)>0\right\}$$
- Averaged Secure Throughput ${\bar{T}}_{u,c}^{\left[\sec\right]}$. The average secure throughput is defined as the difference between the capacity of the legitimate link $C_{u,c}$ (between UE and BS) and the capacity of the eavesdropper $C_{E}$ averaged over the entire area
  (7) $${\bar{T}}_{u,c}^{\left[\sec\right]}=\frac{1}{A}\sum_{(x,y)\in A}\max\left\{0,C_{u,c}-C_{E,c}\left(x,y\right)\right\}$$

### 3.2. Proposed Utility

We define here a new utility that takes into account both security and power consumption. In particular, we define the secure energy efficiency ($EE^{\left[SEC\right]}$).

(8) $$EE^{\left[sec\right]}=\sum_{u,c}EE_{u,c}^{\left[sec\right]}=\frac{\sum_{u,c}\gamma_{u,c}\;{\bar{T}}_{u,c}^{\left[\sec\right]}}{P_{tot}}$$

where

- $\gamma_{u,c}$ is the element (u,c) of the allocation matrix Γ, whose value is
  (9) $$\gamma_{u,c}=\left\{\begin{array}{cc} 1 & \mathrm{if}\;\mathrm{user}\;u\;\mathrm{is}\;\mathrm{associated}\;\mathrm{with}\;\mathrm{cell}\;c\\ 0 & \mathrm{otherwise} \end{array}\right.$$
- $EE_{u,c}^{\left[sec\right]}$ is the energy efficiency of the *u*-th user served by the *c*-th cell.
- $P_{tot}$ is the total power consumption of the network considering both active and idle cells.

Let us define $\Phi =[\Phi \left(1\right),\cdots \Phi \left(c\right),\cdots \Phi \left(N_{C}\right)]$ the vector of length $N_{C}$ (i.e., the number of cells in the area), whose element Φ(c) is one if the *c*-th cell is active, zero otherwise. Consequently, $P_{tot}$ can be written as

(10) $$P_{tot}=\sum_{c=1}^{N_{C}}\Phi \left(c\right)\left(P_{0}+\Omega_{p}P^{\left[\mathrm{TX}\right]}\right)+\left(1-\Phi \left(c\right)\right)P_{idle}$$

**Figure 2:**
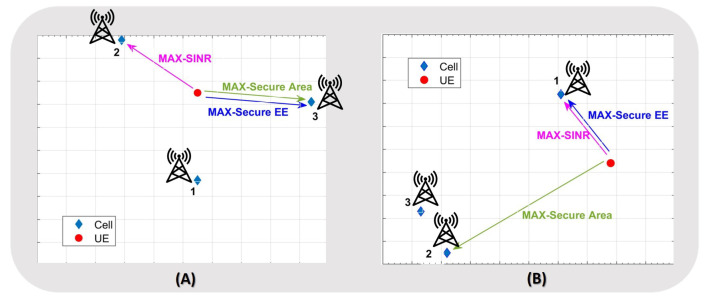
Example of user-cell association based on different metrics.

Given a pattern of active cells, Φ, and an association matrix, Γ, it is possible to calculate the SINR as well as the secure area, the average secure throughput and the proposed utility (8), provided by that association between UEs and cells. As an example, let us suppose to have one user and three cells as in Figure 2. Depending on which cell the user is associated with, different SINR, secure area, and secure EE values can be obtained. The user selects the cell that maximizes the selected utility. For example, in Figure 2, in the case (A) the UE selects the cell-2 if the SINR must be maximized, or the cell-3 if the secure area or the secure EE must be maximized. Differently, in case (B) the cell-1 is selected if the considered utility is the SINR or the EE, while the cell-2 for the achieving the maximization of the secure area.

## 4. Problem Formulation and Solution

### 4.1. Problem Formulation

The goal was to maximize $EE^{\left[\sec\right]}$ defined in (8) over all possible combinations of active/idle cells, Φ, and consequent possible allocation matrices, Γ.

(11) $$\begin{array}{cc} \left(\Gamma^{*},\Phi^{*}\right) & =\max_{\Gamma ,\Phi }\left\{EE^{\left[\sec\right]}\right\}\\ s.t.: & \sum_{c}\gamma_{u,c}=1\;\forall u=1,\cdots ,N_{U} \end{array}$$

The constraint indicates that each user is associated with one serving SBS.

### 4.2. Problem Solution

If the number of users and cells is limited, the optimum solution can be achieved with an exhaustive search (ES).

However, its complexity significantly increases with $N_{C}$ that in a UDN can be very high; thus, we propose an heuristic based on an iterative procedure. We suppose to start with all the cells in idle state, then successively the procedure turns on cells until an increase in the utility function is achieved. More in detail, at the *i*-th iteration, successively, one idle cell (the *c*-th) at a time is activated and the utility function is calculated $EE^{\left[\sec\right]}\left(c,i\right)$. The cell, ${\hat{c}}^{i}$, that provides the highest utility is selected, $EE^{\left[\sec\right]}\left({\hat{c}}^{i},i\right)$. If the new utility value is higher than that at previous iteration (i.e., $EE^{\left[\sec\right]}\left({\hat{c}}^{i},i\right)$>$EE^{\left[\sec\right]}\left({\hat{c}}^{\left(i-1\right)},\left(i-1\right)\right)$), the selected cell is activated. The iteration stops when the utility derived at iteration *i* is lower than that at previous iteration or when all the cells have been activated (i.e., $i=N_{c}$). The procedure is described in Algorithm 1.
**Algorithm 1** Iterative Algorithm of the heuristic procedureInitialization$\Phi^{0}=\left[0,\cdots ,0\right]$  i=1  *Iterations*  **while **$i<=N_{C}$** do** **for** $c=1:N_{C}$ **do**
  $\Phi_{tmp}^{i}=\Phi^{\left(i-1\right)}$    **if** $\Phi_{tmp}^{i}\left(c\right)==0$ **then**
   $\Phi_{tmp}^{i}\left(c\right)==1$     Calculate the allocation matrix that maximizes for each UE the secure EE   Calculate the Utility function (8) $EE^{\left[sec\right]}\left(c,i\right)$    **end if**  **end for**  find ${\hat{c}}^{i}$ s.t. $EE^{\left[sec\right]}\left({\hat{c}}^{i},i\right)=max_{c}\left\{EE^{\left[sec\right]}\left(c,i\right)\right\}$   **if** $EE^{\left[sec\right]}\left({\hat{c}}^{i},i\right)>EE^{\left[sec\right]}\left({\hat{c}}^{\left(i-1\right)},\left(i-1\right)\right)$ **then**
  i=i+1   **else**
  i=C   **end if** **end while**

### 4.3. Computational Complexity

In this section, the complexity of the problem is analyzed.

The problem is deciding which cells must be activated in order to maximize the secure EE. Then the users association becomes straightforward: once the set of active BS is known, each user selects the BS that provides the highest secure EE. The problem is a 0/1 non linear programming problem where the optimization variables are the elements of the association matrix Γ and of cells activation vector Φ.

The problem of deciding which cells must be activated is NP-hard as shown in [34]. Indeed, by assuming an homogeneous scenario, where all cells transmit with the same power, our problem is similar to the one presented in [34] even if the optimization goals are different. In particular, in [34], the goal was to find the minimum set of BSs that guarantees the the minimum achievable rate to all users. Here, we wanted to find the minimum set of BSs (i.e., if the BSs transmit with equal power this corresponds to the minimum consumed power) that provides the highest secure EE. In [34], it has been shown that this kind of problem is NP-hard. Moreover, in general, our problem is more complex, because we consider a heterogeneous scenario with different transmission powers of cells, thus we have higher degrees of freedom.

In particular, if we consider the ES solution, we have that for each possible configuration of the vector Φ, the allocation matrix that provides the maximum secure EE for each user (i.e., $EE_{u,c}^{\left[sec\right]}$) must be derived. Then, among all the possible values of the vector Φ, the one that provides the maximum utility, $EE^{\left[sec\right]}$, is selected. Unfortunately, in UDNs, often this search requires too high complexity. Indeed, the vector Φ can assume $2^{N_{C}}-1$ possible configurations (assuming that at least one BS is active). Hence, the utility function must be calculated $\left(2^{N_{C}}-1\right)$ times.

Differently, if we consider the proposed heuristic, the computational complexity is significantly lower. At the *i*-th iteration the algorithm calculates $\left(N_{C}+1-i\right)$ times the utility function. In the worst case (i.e., all cells are activated and $N_{C}$ iterations are performed) the complexity of the procedure is the calculation of the utility function $\sum_{i=1}^{N_{C}}\left(N_{C}+1-i\right)=\frac{N_{C}\left(N_{C}+1\right)}{2}$ times. However, we have to stress that in a dense environment the number of activated cells is lower than the maximum; hence, in actual system the number of iterations is lower than $N_{C}$ and, hence, the complexity is significantly lower. This is shown in the numerical results section.

## 5. Numerical Results

This section presents the numerical results of the proposed association scheme. In particular, the behavior of the new association policy is investigated either considering the optimal association or the proposed heuristic. To derive numerical results we have considered the parameters listed in Table 2. For what concerns the number of cells and users, we refer to the values of the PPP distribution (i.e., ${\bar{N}}_{C}=\lambda_{S}A$ and ${\bar{N}}_{U}=\lambda_{U}A$). In order to have results not depending on the specific cells deployment, the numerical results have been averaged on several realizations of a given scenario. More in detail, for a given scenario the values of $\lambda_{S}$ and $\lambda_{U}$ are fixed, but in each realization of the scenario users and cells are randomly placed. Consequently, performance of a particular scenario realization is dependent on the particular position of cells and users. In order to avoid this, we have generated 500 different realizations of the same scenario and we have averaged the obtained results.

In order to verify the effectiveness of the proposed association metric and heuristic, different benchmarks have been considered. In particular, the optimal Φ vector and Γ matrix are derived for:

- **Max-SINR** association—each user is associated with the active BS that provides the highest SINR with the goal of maximizing the mean secure throughput per user;
- **Max-Secure Area (SA)** association [29]—each user is associated to the active BS that provides the highest secure area with the goal of maximizing the mean secure area per user;
- **Max-SINR AllOn** association—all the BSs are active, and each user is associated with the BS that provides the highest SINR.

For what concerns the newly defined association metric, we have considered the optimal association (Max-$EE^{\left[sec\right]}$) obtained with the ES and the proposed heuristic (Proposed-Heur). The optimal solution allows to verify the accuracy of the proposed approach, that results to be needed in high dimension scenarios when the ES presents an excessive complexity.

The metrics that are considered here are the secure area and secure throughput averaged per user, the secure EE (i.e., the proposed utility) and the total consumed power.

### 5.1. Without Macrocell

**Figure 3:**
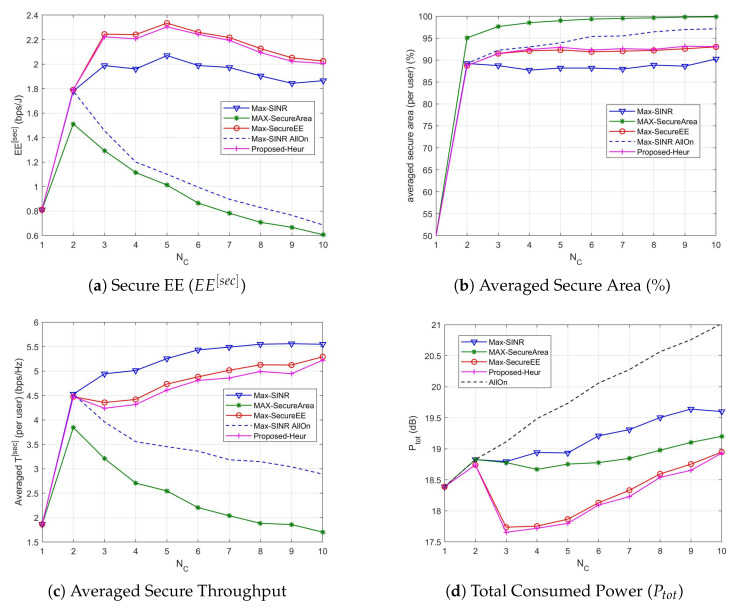
Association performance vs. number of small cells ${\bar{N}}_{C}$ when ${\bar{N}}_{U}=30$.

We start considering the case of having users served only by the small cell layer (i.e., the macrocell is not considered). First of all we show the previous metrics as a function of the mean number of cells when ${\bar{N}}_{U}=30$ in Figure 3.

As we can see, the proposed association policy allows to significantly reduce the power consumption while maintaining a good level of security (both in terms of secure area and secure throughput). In particular, when only 1 or 2 cells are active, the microcell is active (i.e., one microcell is always present in the scenario), for all methods, thus the consumed power is comparable. When the number of cells increases the Max-SecureEE optimization procedure tends to turn off the high power cell and to activate cells with lower power. The power slightly increases with the number of cells (for $N_{C}\geq 3$) to not reduce the throughput, thus achieving the highest EE. The Max-SINR association policy requires higher transmission powers thus achieving the highest secure throughput. The Max-Secure Area method has a power consumption that is in the middle, but it has the lowest secure throughput and EE. For all methods, the power consumption obviously increases with the number of cells in the area (i.e., also the cells in idle mode contribute to the power consumption). In case all the cells are active, obviously the power consumption is the highest and we can observe that the high interference generated by cells increases the security in terms of secure area, but the throughput is reduced due to the SINR reduction. Finally, we can observe that the proposed heuristic is a very good approximation of the optimal Max-secure EE association.

**Figure 4:**
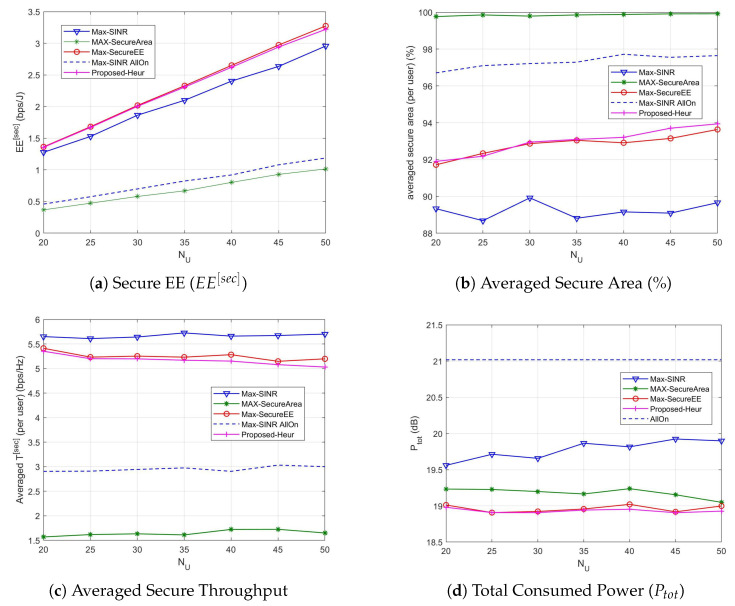
Association performance vs. number of users ${\bar{N}}_{U}$ when ${\bar{N}}_{C}=10$.

In Figure 4 the performance metrics described before are provided when ${\bar{N}}_{U}$ varies and ${\bar{N}}_{C}=10$. As it can be observed from Figure 4b–d, when the number of users increases the power consumption as well as the averaged secure throughput per user and averaged secure area per user do not change significantly. The secure area slightly increases for the proposed utility (Figure 4b), while the power consumption slightly increases for the max-SINR method (Figure 4d). The secure EE method improves the performance when the number of users increases, outperforming all the other methods (Figure 4a). The heuristic algorithm is always able to produce solutions whose quality is very close to that of the solutions returned by the exhaustive search, leading us to propose it as a lower complex method with similar performance. The secure EE is summed over all the users, hence it increases with ${\bar{N}}_{U}$.

### 5.2. With Macrocell

**Figure 5:**
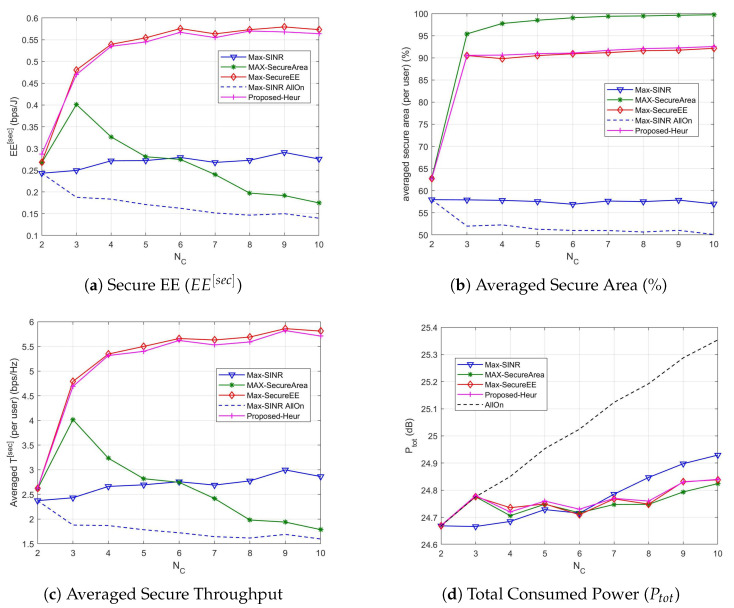
Association performance vs. number of cells ${\bar{N}}_{c}$ when ${\bar{N}}_{U}=30$ (with an always-active macrocell).

We have also considered a second case: users can associate not only with the small-cell layer but also with the macrocell that is placed in the center of the area and is always active (but operates on a different frequency band). The results are presented in Figure 5 when the mean number of small cells varies and the mean number of users is $\bar{N_{U}}=30$. In this case benefits of the proposed association metric are more evident. Indeed, as we can observe it allows to find the best trade-off between different behaviors. With the Max-SINR approach users tend to select the macrocell as serving BS, thus they do not take into consideration security that instead is provided by a suitable exploitation of the intra-layer interference that characterizes the small-cell layer. Consequently, users achieve poor performance also in terms of secure-throughput. The opposite occurs with the Max-Secure Area approach. In this case, the users tend to select the small cells as serving ones, because the intra-layer interference allows a higher protection of communications. This results in very poor performance in terms of secure throughput and EE, because SINR values are quite poor. The proposed method permits to achieve a good trade-off, indeed the secure area is very close to that of the Max-Secure Area method, while it presents a significantly higher secure throughput and EE. In terms of consumed power being the macrocell always active in any case, we cannot appreciate significant differences among different methods. Indeed the macrocell power is significantly higher than the others.

**Figure 6:**
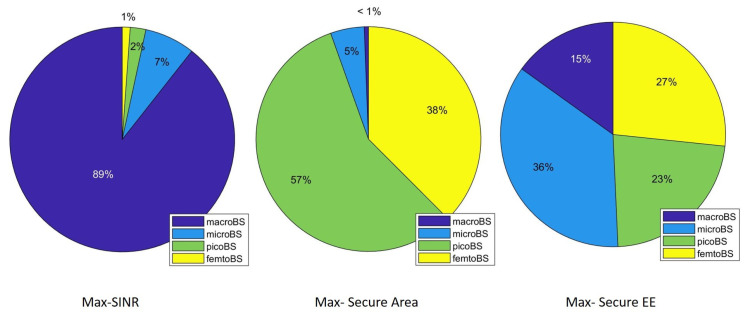
Percentage of association of users with different cells’ type with ${\bar{N}}_{C}=10$ and ${\bar{N}}_{U}=30$.

For supporting the results’ behavior described before, Figure 6 presents the average percentage of users that are connected with different types of cells for the considered association policies. We can see that while Max-SINR tends to associate users with the macrocell, the Max-Secure Area does the opposite. The proposed association metric provides a trade-off between the previous two.

Finally, we want to show that the number of activated cells is usually lower than the maximum, thus significantly reducing the computational complexity of the proposed approach as stated before. Toward this goal Table 3 reports the mean number of activated cell per type in a scenario with ${\bar{N}}_{C}=10$ and ${\bar{N}}_{U}=50$ considering the macrocell or not.

## 6. Conclusions

This paper focused on a ultra dense network where users are under the coverage of multiple cells, thus network performance is strongly influenced by the cell association criterion. In particular, the paper presented a new association policy, where energy efficiency is jointly considered together with communication security. Exploiting the physical layer security it is possible to define a new metric called *secure energy efficiency*

## Figures and Tables

**Table 1:** Power model parameters for different base stations (BSs).

BS Type	$P_{\max}$ [W]	$P_{0}$ [W]	$P_{\mathrm{idle}}$ [W]	$\Omega_{p}$
Macro	20.0	130.0	75.0	4.7
Micro	6.3	56.0	39	2.6
Pico	0.13	6.8	4.3	4.0
Femto	0.05	4.8	2.9	8.0

**Table 2:** Parameters used to produce the numerical results.

Area (*A*)	100×100 mt
${\bar{N}}_{C}$	[1–15]
${\bar{N}}_{U}$	[10–50]
($\alpha_{1}$,$\alpha_{2}$)	(2,4)
$\bar{d}$	25 mt
No. of Macro BS	m=0/1
No. of Micro BS	1
No. of Pico BS	50$\%\;(N_{C}-1-m)$
No. of Femto BS	50$\%\;(N_{C}-1-m)$
$P_{macro}^{\left[\mathrm{TX}\right]}$	20 W
$P_{micro}^{\left[\mathrm{TX}\right]}$	5 W
$P_{pico}^{\left[\mathrm{TX}\right]}$	0.13 W
$P_{femto}^{\left[\mathrm{TX}\right]}$	0.05 W

**Table 3:** Mean number of activated cells when $\bar{N_{C}}=10$ and $\bar{N_{U}}=50$.

**Without Macrocell**			
**Cell Type**	**Max-SINR**	**Max-Area Sicura**	**Max Secure EE**
Micro	0.8	0.1	0.05
Pico	0.5	1.2	1.1
Femto	0.8	1.3	0.9
**With Macrocell**			
Macro	1	1	1
Micro	0.6	0.2	0.4
Pico	0.5	1.1	0.7
Femto	0.7	1.4	1

## Data Availability

No data available.
